# On the origin of degeneracy in the genetic code

**DOI:** 10.1098/rsfs.2019.0038

**Published:** 2019-10-18

**Authors:** D. L. Gonzalez, S. Giannerini, R. Rosa

**Affiliations:** 1 CNR-IMM, UOS di Bologna, Via Gobetti 101, 40129 Bologna, Italy; 2 Dipartimento di Scienze Statistiche, Università di Bologna, via delle Belle Arti 41, 40126 Bologna, Italy

**Keywords:** genetic code, degeneracy, molecular evolution, symmetry, protein coding

## Abstract

The degeneracy of amino acid coding is one of the most crucial and enigmatic aspects of the genetic code. Different theories about the origin of the genetic code have been developed. However, to date, there is no comprehensive hypothesis on the mechanism that might have generated the degeneracy as we observe it. Here, we provide a new theory that explains the origin of the degeneracy based only on symmetry principles. The approach allows one to describe exactly the degeneracy of the early code (progenitor of the genetic code of LUCA, the last universal common ancestor) which is hypothesized to have the same degeneracy as the present vertebrate mitochondrial genetic code. The theory is based upon the tessera code, that fits as the progenitor of the early code. Moreover, we describe in detail the possible evolutionary transitions implied by our theory. The approach is supported by a unified mathematical framework that accounts for the degeneracy properties of both nuclear and mitochondrial genetic codes. Our work provides a new perspective to the understanding of the origin of the genetic code and the roles of symmetry principles in the organization of genetic information.

## 1. Introduction

Extant genetic codes can be seen as a mapping between two different sets: the 64 possible mRNA codons and the 20 amino acids plus the stop signals needed for protein synthesis. Since the cardinality of the starting set of codons (64) is greater than the cardinality of the arriving set of amino acids (20 + 1), the mapping is necessarily degenerate. In other words, some amino acids are coded by two or more codons. Degeneracy is a concept introduced first in quantum mechanics. An energy level of a quantum system is degenerate if it corresponds to two or more different quantum states with the same energy. Degenerate quantum states are described by different solutions of the Schröedinger equation linked by a symmetry transformation; that is to say, quantum degeneracy is essentially a consequence of symmetry. Thus, it is natural to ask if the degeneracy of the genetic code can also be related to symmetry properties. Even though many theories on the origin of the genetic code and protein coding have been put forward, the origin of degeneracy remains a very elusive problem.

Symmetry is a meta-principle that pervades all the branches of physics: from classical mechanics to quantum theory, from relativity to particle physics, it is common knowledge that symmetry principles are invoked to explain conservation laws. For instance, in classical mechanics, energy conservation is related to the Hamiltonian invariance under time translation; also, the conservation of linear and angular momenta is related to the Hamiltonian invariance under space translations and space rotations, respectively. In accordance with the universal role played by symmetry principles in physics, we think that this approach can contribute to understand some important unsolved biological problems.

Several attempts to describe the genetic code in terms of symmetry properties and group theory have been developed [1–3]. Note that this approach has not always been received well by biologists, maybe because of the difficulty of providing a biological interpretation to models that resort to an improbable chain of symmetry breaking steps; in this respect, the criticism by Maddox [4] that regards these efforts as a ‘valuable exercise in classification’ is well posed. However, we argue that the analysis based on the symmetry properties is fundamental to the comprehension of the origin and structure of the genetic code. In this work, we describe the origin of degeneracy by using a new approach based on symmetry that allows an exact quantitative description of some fundamental features such as the degeneracy distribution. Among the few works that address the study of degeneracy by using symmetry properties are [5–7]. For further works about symmetry and symmetry breaking in the genetic code, see among others [8–14].

It is undeniable that the present form of the genetic code is at least in part due to historical accidents. We can ask ourselves: ‘If life on earth would originate again, then what would be the structure of the protein synthesis apparatus (assuming that there would be one)? Would there be a universal genetic code, exhibiting degeneracy?’ Apparently, no one can answer this questions but it is plausible to assume some structural similarities with the extant apparatus due to the constraints imposed by the universal laws of chemistry and physics that continue to hold.

Theories on the origin of the genetic code can be grouped in at least five different categories. (1) The stereo-chemical origin: it is based on the hypothesis that codons (or also anti-codons) can selectively bind to assigned amino acids via a stereo-chemical specificity [15,16]. Note that a correlation between some amino acids and aptamers containing codon/anticodon motifs has been found in [17] and this brings some support to such hypothesis. (2) The co-evolution theory: it postulates that the codon assignation to new amino acids proceeds by inheriting part of the codon set pertaining to the precursor amino acids (amino acids that generate the new one by biosynthetic modification) [18,19]. See also [20] for the temporal order of amino acids. (3) The adaptive hypothesis: it postulates that the main evolutionary pressure is minimization of mutation errors; moreover, it implies that similar amino acids are coded by similar codons [21]. (4) The operational code: it proposes an ancestral link between the operational code (which determines mainly in the acceptor stem the affinity with the cognate amino acid) and the genetic code (implemented with the codon–anticodon pairing) [22–25]. (5) The frozen accident: it postulates a random origin of the codon assignation to amino acids and a successive evolution due to different evolutionary pressures, until a point in which any further modification becomes deleterious (determining the freezing of the code) [16]. Remarkably, none of the approaches described above is centred on the degeneracy distribution as a key feature. Degeneracy is more a consequence than a property directly related to the physico-chemical origin of the code. We note two exceptions, i.e. [5,7], the latter, however, refers to a biochemical explanation of degeneracy for extant codes, and thus, is not directly related to the evolution of the code

The evolutionary path of the genetic code can be partitioned into two main periods, i.e. the ancient period, from the beginning to the appearance of the universal genetic code of LUCA (the last universal common ancestor), and the modern period, from this universal genetic code onwards [26]. For a recent discussion on the definition of LUCA, see [27]. According to [7], the ancient period can be subdivided further by including a third, intermediate, period situated immediately before the comparison of the genetic code of LUCA. In this phase, the code underwent some kind of optimization through the development of post-transcriptional modifications in the first base of anticodons. In [7], a description of the post-transcriptional modifications that characterize the genetic code of LUCA is proposed. As concerns extant codes, in [5] a biochemical explanation of the wobble hypothesis is provided by studying the stability of the second letter of the anticodon at the ribosome centre. Note that in both [5] and [7] the regularity of the genetic code known as Rumer’s transformation represents a key aspect. It is a global symmetry of the genetic code discovered by the theoretical physicist Y. Rumer in the 1960s [29].^1^ Notably, Rumer’s symmetry can be described exactly as a dichotomic class in terms of the chemical characters of the first two nucleotides of a codon [31–33].

In our approach, we extend back the fundamental connection between symmetry and degeneracy to the ancient evolutionary period of the genetic code. To this aim, we develop a model of the putative ancestor of the genetic code of LUCA. In the literature, there is some agreement about the fact that the degeneracy distribution of this ancestor genetic code should coincide with that of the present vertebrate mitochondrial genetic code [7,34]. In our view, a satisfactory model of the early code needs to possess: (i) the exact quantitative description of the degeneracy distribution and (ii) the fundamental Rumer’s symmetry which is inherited by the LUCA’s and subsequent codes. We build a model that satisfies these two criteria, based on stereo-chemical symmetries of ancient chemical molecules and their informational counterpart as sequences of nucleotides.

In the first part of the work, we describe in detail the model. Surprisingly, the first criterion mentioned above can be satisfied only if we consider a special set of 64 four-base codons, i.e. tesserae (from the greek tessera = four), and a set of ancient symmetric adaptors [35] with anticodons of the same length. The solution provided by the model is unique and the description of the degeneracy of the early code implies the use of codons of length four. This qualifies the tessera code as a putative ancestor of the early genetic code, i.e. a pre-early genetic code. This complies with the hypothesis that the code originated with codons longer than three nucleotides [36]. We dedicate the second part of the work to analyse the plausibility of the evolutionary transitions implied by the model.

## 2. Symmetry and degeneracy

**Figure 1.**
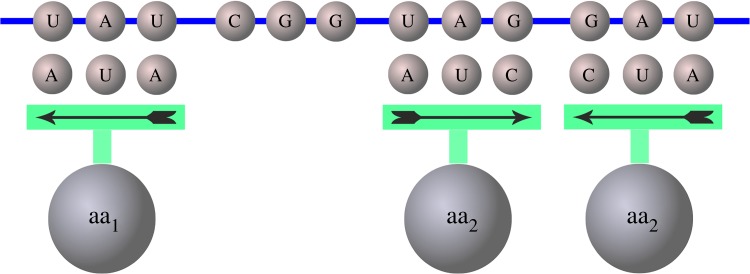
Schematic representation of the decoding through primeval reversibile tRNA adaptors that can read codons in both directions. A tRNA with anticodon AUA (left) can be paired only with the codon UAU, whereas a tRNA with anticodon AUC (right) can be paired with codons GAU and UAG. Hence, the amino acid aa_1_ carried by the first tRNA will have degeneracy 1 and that carried by the second tRNA (aa_2_) will have degeneracy 2. (Online version in colour.)

In order to show the connection between degeneracy and symmetry consider hypothetical reversible tRNA adaptors that follow a Watson–Crick-like pairing rule (no wobble position); see figure 1. Note that we require only that recognition of tesserae–antitesserae be performed by complementary pairing. In this respect, it is not essential that the chemical binding be strictly Watson–Crick. Indeed, in present forms of mRNA and tRNA, Watson–Crick pairs do not allow to recognize codons in the reverse direction. However, in pre-LUCA times, analogue ancient molecules could have allowed a bi-directional recognition. For example, it has been proposed that the first genetic material used a simpler backbone than ribose [37]. For such molecules, the pairing strand direction is probably not as constraining as in actual DNA/RNA molecules. In [38], it has been suggested that nucleic acids where R-ribose has been replaced by L-ribose may hybridize with natural DNA and RNA and adopt a parallel-stranded A form. In extant organisms, specific nucleotide sequences can adopt a parallel orientation that involves non-canonical base pairing. In particular, parallel-oriented regions have been found in bacterial (*Escherichia coli*, *Listeria innocua*) and insect genomes (*Drosophila melanogaster*); such unusual structures are postulated to have a remarkable evolutionary role, and a significant impact on biological processes [39].

Another fact supporting the ‘reverse recognition’ hypothesis is that a codon and its reverse codon always code for similar amino acids [40], and in [6] this has been proposed as a sort of a relic of such process. Another interesting possibility, put forward in [6], is that ancient adaptors, like pre tRNAs, lacked the D and T loops; hence, being almost symmetric, they could have been able to bind in both directions. The reversing symmetry of ancient adaptors implies that they can recognize codons both in the 5′–3′ and 3′–5′ direction. Such possibility has been explored in a different context for explaining the origin of the genetic code with reversible primeval adaptors that read only two of the three bases of the codon and include some sort of wobble pairing [6,28]. Remarkably, reversibility of the adaptors induces naturally a form of degeneracy in codon recognition. For instance, a tRNA with anticodon AUA can be paired only with the codon UAU. Instead, a tRNA with anticodon AUC can be paired with codons GAU and UAG. Hence, the amino acid carried by the first tRNA will have degeneracy 1, whereas that carried by the second tRNA will have degeneracy 2 (figure 1). In general, tRNAs with palindromic anticodon (i.e. invariant with respect to inversion) code amino acids of degeneracy 1, while non-palindromic anticodon code for amino acids of degeneracy 2. Counterintuitively, the more symmetric an anticodon the less degenerate the associated amino acid.

Based upon the arguments described above, we show how to build a model of the degeneracy of the early code. First of all, it is straightforward to show that if the codons have length three, then the problem has no solution. In fact, we would obtain 16 symmetric (palindromic) codons associated with 16 amino acids with degeneracy 1 and 48 codons with no symmetry, associated with 24 amino acids with degeneracy 2. Clearly, these degeneracy values (either 1 or 2) do not match the degeneracy values of the early code (i.e. 2 or 4). The degeneracy values can be augmented by considering more symmetries. The main symmetries naturally associated to DNA or RNA molecules are the reverse, complementary and reverse-complementary ones (plus the trivial identity).^2^ These symmetries appear in real sequences, for example, through inversions and inverted transpositions, and might be responsible for the genomic balance known as the second Chargaff rule [41].^3^ Now, if we consider adaptors having both the reverse and the reverse-complementary symmetries, then we obtain the following degeneracy distribution: 16 codons associated with eight amino acids with degeneracy 2, and 48 codons associated with 12 amino acids with degeneracy 4. This matches the degeneracy values of the early code (i.e. 2 and 4) but the degeneracy distribution is different: 32 codons associated with 16 amino acids with degeneracy 2, and 32 codons associated with eight amino acids with degeneracy 4. Note that we have eight amino acids with degeneracy 2, whereas the early code has 16 amino acids with degeneracy 2. This means that the model needs 32 symmetric codons (not just 16) and there is only one way to achieve the goal, namely, consider codons with more than three nucleotides.

**Figure 2.**
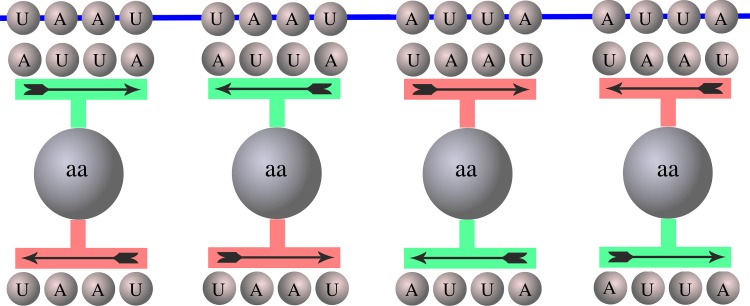
Schematic representation of the tessera decoding through primeval adaptors that possess two palindromic and self-complementary anticodons. We show a single adaptor that carries the anticodons AUUA and UAAU in the four possible pairing configurations. Since the anticodons are palindromic only two different tesserae can be read, so that the cognate amino acid will have degeneracy 2. (Online version in colour.)

The exact, unique solution to this problem is provided by the tessera code (table 1), a special set of 64 four-base codons that are recognized by a set of ancient adaptors possessing both the palindromic and the reverse-complementary symmetries (figure 2). We build the tessera set of 64 length-four codons by exploiting symmetry properties related to group theory; consider the four symmetric transformations of the bases: Identity (I: (A,U,C,G) → (A,U,C,G)); Strong/Weak or complementary (SW: (A,U,C,G) → (U,A,G,C)), Pyrimidine/Purine (YR: (A,U,C,G) → (G,C,U,A)) and Keto/Amino (KM: (A,U,C,G) → (C,G,A,U)). This set of transformations *F* = {I, SW, YR, KM} (together with the composition operator) is isomorphic to the Klein 4-group of symmetry (see electronic supplementary material, A). We start from the four single nucleotides: {A,U,C,G}; we apply the four transformations and append the resulting nucleotides to the original one. For instance, starting from A and applying *F* = {I, SW, YR, KM} one obtains {AA, AU, AG, AC}. This first step produces the 16 possible dinucleotides. Now, we apply again the four possible transformations to the 16 dinucleotides and append them to the original ones. For instance, starting from AU and applying *F* = {I, SW, YR, KM}, one obtains {AUAU, AUUA, AUGC, AUCG}. This second step produces the tessera set of 64 length-4 objects. From a mathematical point of view, a tessera is a quadruplet *b*_1_*b*_2_*b*_3_*b*_4_ where *b*_*i*_ ∈ {A,U,C,G} and *b*_3_*b*_4_ = *f*(*b*_1_*b*_2_) where *f* ∈ *F*. In table 1, we present the tessera set. It is partitioned in 16 quartets corresponding to the transformations that produce them (reported on the left side of any quartet). Note that 16 tesserae are palindromic (first column), 16 are self-complementary (second column) and 32 have none of these symmetries.

**Table 1.** Complete table of tesserae (four-base codons with symmetry properties). Each of the 16 quartets contains four tesserae and the transformation that generates them acting on the first doublet as to obtain the second doublet. Inside quartets, tesserae with the same colour code for the same amino acid: pink and green = 2 + 2 and white = 4.

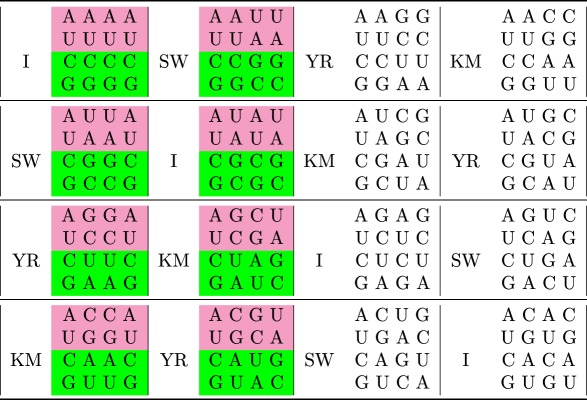

**Figure 3.**
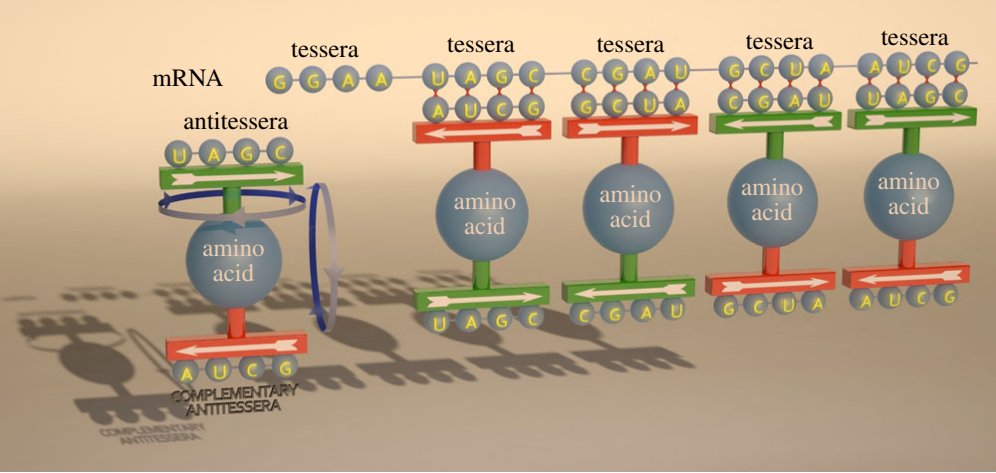
Schematic representation of the tessera decoding through primeval adaptors that possess two non-symmetric antitesserae. The four possible spatial configurations of the adaptor are paired with four different tesserae and produce an amino acid with degeneracy 4. (Online version in colour.)

By reading the set of tesserae through the primeval tRNA adaptors defined above, we can completely explain the degeneracy distribution of the early genetic code. In analogy with the example depicted in figure 1, the degeneracy associated to the tessera decoding depends on its symmetry, namely, the more symmetric an (anti)tessera the less degenerate the associated amino acid. Antitesserae are defined analogously to anti-codons, i.e. the reverse complement of a tessera. tRNAs with palindromic antitesserae can read palindromic tesserae in pairs; this implies that the associated amino acid has degeneracy 2. This is shown in figure 2 where the adaptor, no matter the symmetry applied, can read only the pair of tesserae AUUA, UAAU. Since there are 16 palindromic tesserae, this decoding strategy produces eight amino acids of degeneracy 2. The same argument holds for the 16 self-complementary tesserae and this produces eight additional amino acids with degeneracy 2. Now, a tRNA with a non-symmetric antitessera can read four different non-symmetric tesserae (third and fourth columns of table 1). In turn, this implies that the eight amino acids attached to these tRNAs have degeneracy 4. Figure 3 shows this case where a non-symmetric antitessera is paired with four different (non-symmetric) tesserae. Overall, we obtain 16 amino acids with degeneracy 2 and 8 amino acids with degeneracy 4. This is, in fact, the actual degeneracy distribution of the putative early code, and coincides with that of the present vertebrate mitochondrial code (where degeneracy-6 amino acids contribute inside any quartet with two groups of codons, one group with degeneracy 2 and the other with degeneracy 4). In table 2, we report the number of codons, number of symmetric codons and the different degeneracy distributions as a function of the codon length. Note that the 4T solution (tesserae of length 4) is the only case that gives the degeneracy of the vertebrate mitochondrial genetic code.

**Table 2.** Number of codons, number of symmetric codons and degeneracy distribution as a function of the codon length. Note that the 4T solution (tesserae of length 4) is the only case that gives the degeneracy of the vertebrate mitochondrial genetic code.

codon length	no. codons	no. symmetric codons	degeneracy distribution	
			degeneracy	no. aa
2	16	8	2	4
			4	2
3	64	16	2	8
			4	12
4	256	32	2	16
			4	56
4T	64	32	2	16
			4	8
5	1024	64	2	32
			4	240

A strong support to our hypothesis on the origin of degeneracy is provided by a mathematical model of the genetic code that explains the degeneracy of both the nuclear and the mitochondrial variants. The model, described in [33,42], is based on number theory, i.e. redundant integer number representation systems (see box 1). Usual numeration systems are based on the additive decomposition of a number using the powers of a base *b*. In this case, each number has a unique representation. On the contrary, non-power numeration systems use a sequence that grows more slowly than the powers of a base. In the latter instance, a number can have a non-unique (degenerate) representation and this can be used to describe the degeneracy distribution of the genetic code. The analysis of the euplotid nuclear genetic code leads to the unique solution: 8, 7, 4, 2, 1, 1; see box 1 and [42]. The same analysis for the vertebrate mitochondrial genetic code leads to the unique solution: 8, 8, 4, 2, 1, 0; see box 1. In particular, this last result implies the partition of the code in two equivalent sets. Each set can be interpreted in terms of dinucleotides, and the complete representation as merged pairs of dinucleotides. In this way, exactly as in our biological hypothesis, codons of four nucleotides arise naturally. For a detailed description of the genesis of the tessera set and its connections with the non-power model of the genetic code, see [33,35].

Box 1. **Synopsis of non-power numeration systems and their application to modelling the nuclear euplotid and vertebrate mitochondrial genetic codes.**

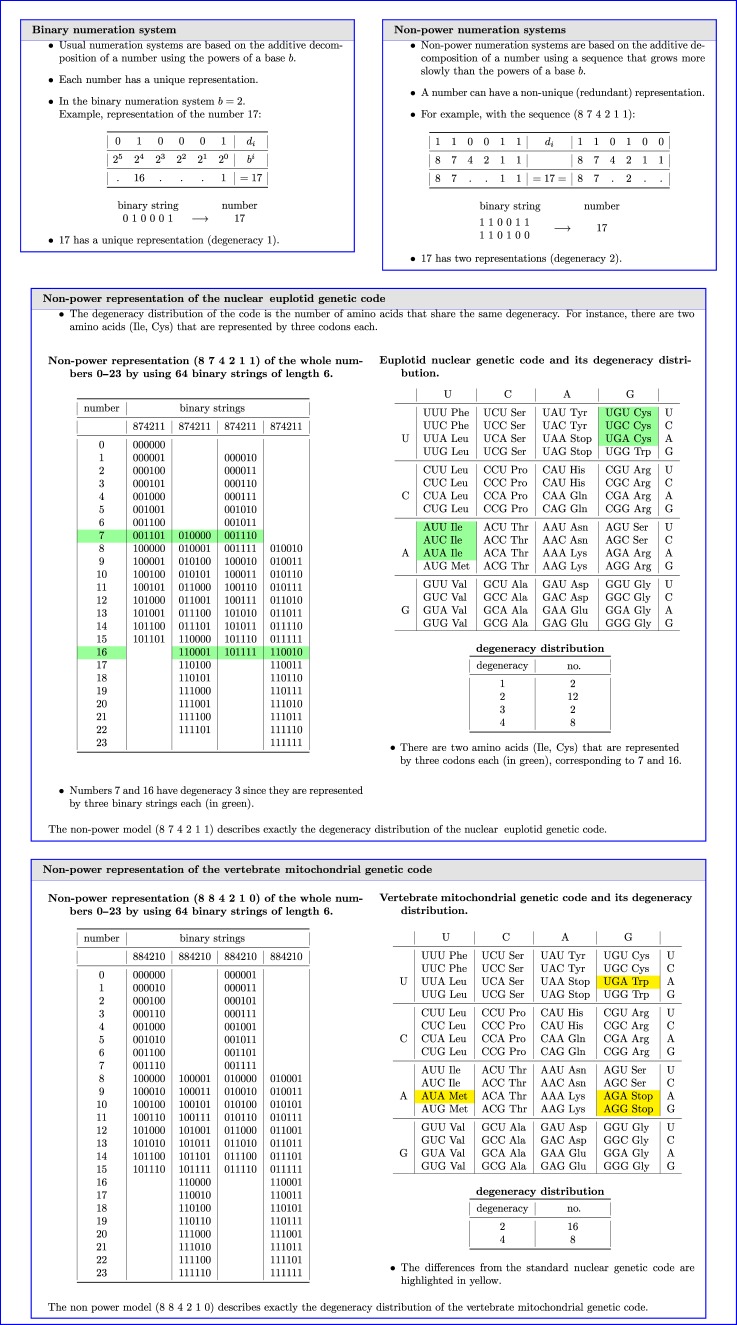

## 3. Evolutionary implications

**Figure 4.**
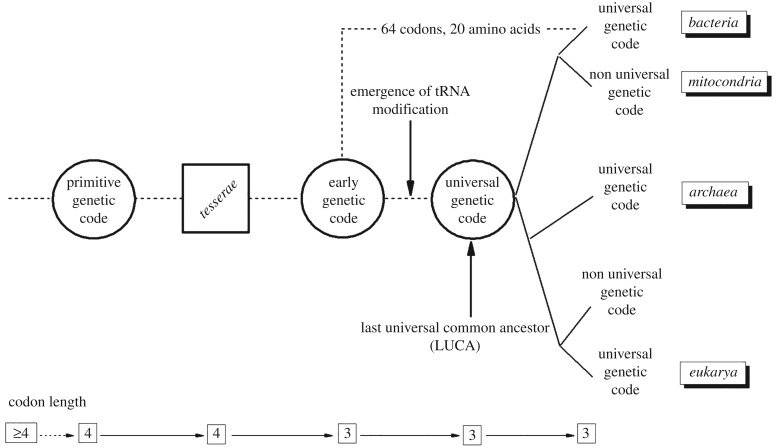
Representation of the evolution of the genetic code, adapted from [34]. Each circle or square represents a milestone. The bottom line shows the evolution of the codon length implied by our theory.

To the best of our knowledge, the tessera code represents a first quantitative explanation for the origin of degeneracy in ancestral codes. As such, it might be relevant for explaining the evolution of the genetic code. In the second part of this article, we analyse the possible evolutionary implications of the tessera code. To this aim, we recall the evolutionary hypothesis presented by Watanabe & Yokobori [34] which is based on the analysis of translation in extant mitochondria (see figure 4 adapted from [34]). Figure 4 presents milestones on which there is some agreement and that represent the evolutionary steps from a primitive genetic code to the present variants. Starting from extant variants and going backwards in time we find the first milestone, i.e. the universal genetic code of LUCA. This code is hypothesized to have a structure similar to that of the present nuclear standard genetic code. The simplest variant of extant codes is the vertebrate mitochondrial genetic code that, mainly for this reason, has been proposed as a model of the predecessor of the universal LUCA code: the early code (the second milestone from the right in figure 4). The main evolutionary novelty implied by the transition from the early to the universal code is the appearance of post-transcriptional modifications in tRNAs. This is supported by the fact that, in some extant Metazoan mitochondria, an unmodified U at the first position of the anticodon can pair with all the bases at the third position of the codon [43]. This allows to decode families of codons without the need for modified nucleotides (a family is a group of four codons sharing the first two bases and coding for the same amino acid). In the proposal of Watanabe & Yokobori [34], the early code is derived from a primitive code with fewer, more degenerate, amino acids, i.e. Jukes’ code [44]. Such code is supposed to be formed only by families with the exception of one amino acid and the stop signal which have degeneracy two, i.e. are coded by two codons.

Now, our main claim is that the tessera code represents an ancestor of the early code, namely, a pre-early code, placed between the primitive and the early code. There are several arguments that support the tessera code as a pre-early code (the square milestone in figure 4). First and foremost, it has exactly the same degeneracy structure as the early genetic code. Moreover, the tessera code is in agreement with the hypothesis of Baranov *et al*. [36], proposing an origin of the code with long oligonucleotides, followed by a diminution in codon length until the optimal number of 3 was reached. Assuming that the pre-early code has codons of length 4 implies that also the primitive code has codons of length at least 4. Indeed, we show that Jukes’ primitive genetic code can be implemented with generic codons of length 4. In the following description, we use the term codon to mean codons of length four or tetracodons. Jukes’ code has 15 amino acids with degeneracy 4, one amino acid with degeneracy 2 and two stop codons. Overall, there are 15 elements with degeneracy 4 and 2 elements with degeneracy 2. If we assume that this code originated from codons of 4 nucleotides, then this implies the choice of 17 elements/amino acids that can be coded by either two or four codons taken from the set of 4^4^ = 256 codons. Note that the set of 256 codons can be partitioned into a subset of 32 codons that possess some symmetries (these correspond to the first two columns of the tessera code of table 1) and a subset of 224 codons with no symmetry. As shown above, symmetric codons correspond to amino acids with degeneracy 2, whereas asymmetric codons correspond to amino acids with degeneracy 4. Now, if we assume a random mechanism for the assignation of codons to amino acids the degeneracy distribution that has the highest probability corresponds exactly to that of Jukes’ code. We have shown this in the electronic supplementary material B. Jukes assumes that one of the elements of degeneracy 2 is associated with the stop signal. With this choice, stop codons are less prone to be generated by random errors, i.e. these elements are less ambiguous than those with degeneracy 4. Likewise, it is natural to hypothesize that the other element with degeneracy 2 corresponds to the amino acid that codes for the start signal.

We have shown that the primitive code (Jukes’ version) is naturally described with codons of length 4. Now, we describe a possible evolutionary path from Jukes’ code to the tessera code. In our approach, the primitive code is composed of two pairs of symmetric codons and 60 non-symmetric codons. Note that the selection of symmetric codons, due to their diminished propensity to point mutations error, represents a first step for selecting the symmetric half of the tessera set. Suppose that a new adaptor possessing a symmetric anticodon appears and competes (carries the same amino acid) with an existing adaptor having an asymmetric anticodon. The new adaptor can bind to symmetric codons that are part of the tessera set (first two columns of table 1). Such adaptor has an evolutionary advantage over the one carrying a non-symmetric anticodon, because it has two different spatial configurations that can be used for binding with the codon. For example, the adaptor in figure 2 carries the palindromic anticodon AUUA. If the adaptor is reversed, it can still bind to the codon/tessera UAAU. This selective pressure causes the capture of all the symmetric codons (tetracodons)/tesserae at the expense of non-symmetric codons. At the end of this process, we have a code composed of 32 symmetric tesserae, and 32 non-symmetric tetracodons that do not necessarily belong to the tessera set. At this point, a further optimization step is reached by selecting non-symmetric tesserae: as shown in [35], tesserae are immune to point mutations^4^ and, thus, survive to non-tessera tetracodons due to the evolutionary pressure of decoding accuracy. The 32 symmetric tesserae are immune to point mutations (two simultaneous, highly improbable, point mutations are needed to produce a transition between two tesserae). This means that the corresponding tRNAs do not lead to the incorporation of a non-cognate amino acid if they are subjected to a point mutation. This property of error detection implies an evolutionary advantage in terms of accuracy of protein synthesis. The remaining 32 non-symmetric tetracodons are not necessarily tesserae, but those that are tesserae have the property of error immunity so that they will be gradually selected for the above reasons. Hence, we obtain the complete structure of the tessera pre-early code whose degeneracy distribution coincides with that of the early code (and with that of the extant vertebrate mitochondrial code).

We have shown that the transition between Jukes’ code and the tessera code is the most probable under minimal assumptions. Clearly, in this transition, the tessera recognition becomes more specific than the tetracodon recognition of Jukes’ code. Indeed, the property of error detection of the tessera code allows to reduce the ambiguity related to the amino acid loading of tetracodon adaptors. In turn, the increased precision in the tessera recognition allows to refine the choice of amino acids due to the evolutionary pressure of protein performance.

**Figure 5.**
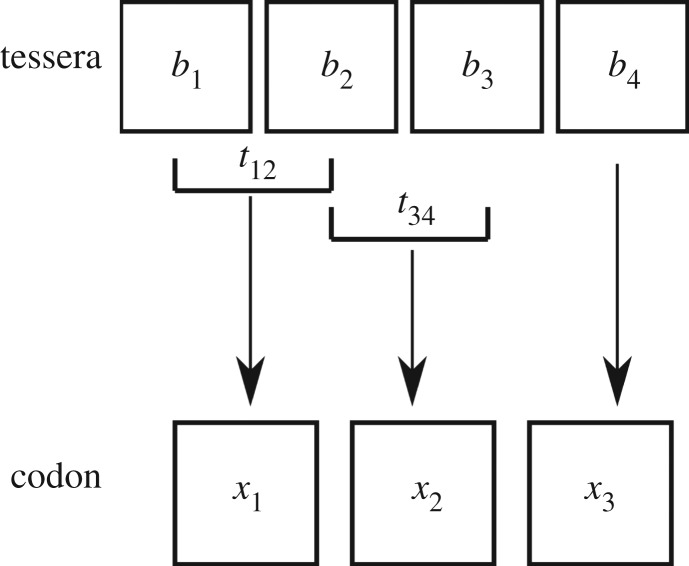
Schematic representation of the mapping between the tessera (*b*_1_ *b*_2_ *b*_3_ *b*_4_) onto the codon (*x*_1_ *x*_2_ *x*_3_).

Our hypothesis of the tessera code as a pre-early code implies also another major evolutionary transition, i.e. the transition between the tessera code and the early code. Since the early code is supposed to have codons of length three, the major problem implied by this transition is related to the change in codon length which is generally considered deleterious [16]. Note that (i) any theory on the origin of the code with codon length different from three must face this problem [36] and (ii) such transition is deleterious when an evolutionary level in which the code has frozen is reached since this implies a dramatic change in the sequence of amino acids of all the proteins of an organism; however, this is not necessarily the case in previous evolutionary steps closer to the origin of the code. The tessera code allows to find a neat solution to the problem of the transition from tetracodons to codons. In fact, the information carried by the tessera set is redundant. By definition, if any three, out of four, letters of a tessera are known, then the missing letter can be derived univocally. Hence, from the point of view of coding theory, the tessera code and any trinucleotide genetic code carry the same informational content. This implies that a one-to-one mapping between tesserae and codons can be established. The essential structure of such mapping entails that the transformations between adjacent letters of a tessera become the nucleotides of a codon. In particular, given a tessera *b*_1_*b*_2_*b*_3_*b*_4_ we can have three chemical transformations between adjacent letters: *t*_12_ = *f*(*b*_1_*b*_2_) between *b*_1_ and *b*_2_, *t*_23_ = *f*(*b*_2_*b*_3_) between *b*_2_ and *b*_3_, and *t*_34_ = *f*(*b*_3_*b*_4_) between *b*_3_ and *b*_4_. Note that only two of these three transformations are independent since *t*_34_ = *t*_12_. In table 4*a*, we have rearranged the tessera code according to the transformation *t*_12_ (rows) and *t*_23_ (columns). We propose that *t*_12_ and *t*_23_ be mapped onto the first and second nucleotide of the codon, respectively (*x*_1_, *x*_2_). This correspondence is shown in table 3. Moreover, the fourth letter *b*_4_ is mapped onto the third nucleotide of the codon *x*_3_. A schematic representation of the mapping is presented in figure 5. Note that, according to this mapping, the columns of the tessera set are mapped onto the columns of the genetic code so that *t*_23_ = I is mapped onto NAN codons (degeneracy non-4), and *t*_23_ = KM is mapped onto NCN codons (composed only of families); compare table 4*b* with table 4*c*. We can observe that these two columns of the tessera code share the same degeneracy with the corresponding columns of the genetic code (either 4 or 2 + 2). The natural completion of the mapping assigns *t*_23_ = SW to NUN codons and *t*_23_ = YR to NGN codons. The latter two assignments need to account for some exceptions determined by the fact that in the transition from tesserae to codons Rumer’s symmetry is indeed preserved but the self-complementary symmetry cannot. The tessera–antitessera interaction is more specific than the codon–anticodon one, due to the presence of four Watson–Crick-like chemical bonds. However, in the case of extant genetic code, the degeneracy is mainly determined by the codon–anticodon interaction of the first two bases. Hence, by assuming that the binding energy in pre-early code times is comparable to the Watson–Crick one, the tessera–antitessera interaction energy should be approximately double the actual codon–anticodon energy.

**Table 3.** Basic structure of the mapping between tesserae and codons. The four transformations between the bases of a tessera are mapped onto the four nucleotides of a codon.

tesserae transformations *t*_12_,*t*_23_		codon bases *x*_1_, *x*_2_
I	⟶12345	A
SW	⟶12345	U
KM	⟶12345	C
YR	⟶12345	G

**Table 4.** (*a*) The tessera code organized according to the transformations: first–second letter *t*_12_ (rows) and second–third letter *t*_23_ (columns); (*b*) the same as (*a*) but with the swapped quartets as indicated by the arrows. (*c*) Degeneracy of the vertebrate mitochondrial genetic code. The codons of the vertebrate mitochondrial code in (*c*) and the tesserae (*b*) are related through the one-to-one mapping described in the text. Inside quartets, tesserae with the same colour code for the same amino acid: pink and green = 2 + 2 and white = 4.

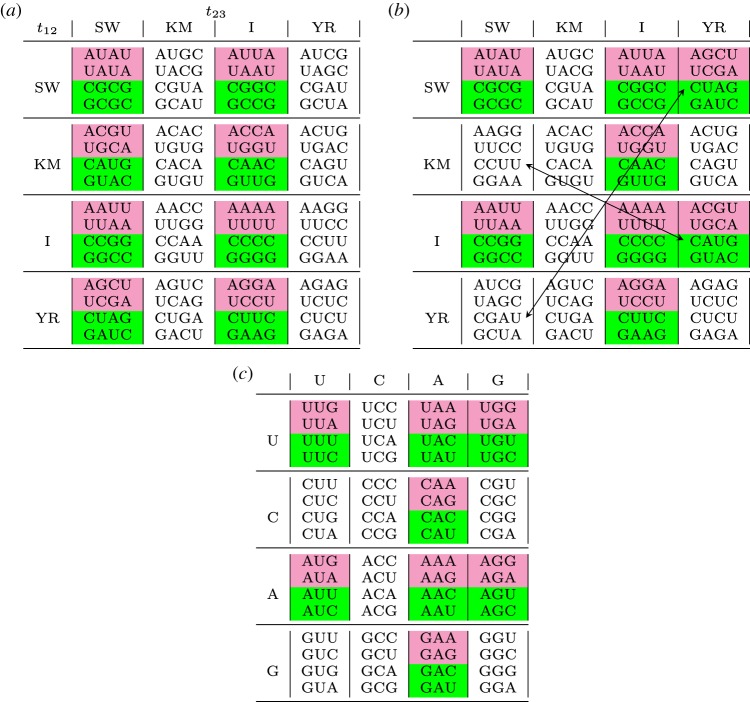

Thus, from a biochemical point of view, the transition from tesserae to codons implies the transition between a full four-base long specific Watson–Crick-like pairing for reading tesserae to the wobble strategy for reading codons.

In particular, this implies theoretical constraints on some symmetry properties that are present in the world of tesserae but are not in extant codes, for instance, the loss of the self-complementary symmetry. Indeed, each column of the tessera code has a definite degeneracy but in extant codes this is true only for two columns, i.e. codons of the kind NMN (NAN or NCN). Instead, the columns corresponding to codons NKN (NUN or NGN) have mixed degeneracy; in particular, the differing quadrants between the two codes are those of the kind SUN and WGN (we call WSN or SWN mixed as opposed to the non-mixed SSN WWN). In other words, extant codes have codons of the kind WGN that codify amino acids with degeneracy 2 despite the fact that the central base is strong, and codons of the kind SUN that codify amino acids with degeneracy 4, despite the fact that the central base is weak [53].

An explanation of such features in terms of energetic constraints depending on the stereo-chemistry of codon–anticodon interaction is proposed in [5]. In the extant genetic code, a weak interaction is normally associated with a 2 + 2 degeneracy. Indeed, this is the case for codons of the kind NAN, AUN and UUN. However, in the case of a U as second letter, a further stabilization of the purine central letter N_35_ in the anticodon loop of the tRNA by U_33_ allows to read a complete family despite the weak character of N_35_.^5^ In the mirror case, for codons of the kind AGN and UGN the nucleotide N_35_ is not sufficiently stabilized by U_33_ and the associated quartet becomes of degeneracy 2 + 2.

These wobble strategy restrictions imply that in the mapping from tesserae to codons the quadrant (YR-SW) is swapped with quadrant (SW-YR) and quadrant (KM-SW) with quadrant (I-YR); see table 4 (upper panels). Eventually, the fourth letter of a tessera is mapped onto the third letter of a codon with the following exception that ensures a correct grouping: if *b*_4_ = K (T or G) then *x*_3_ = KM(*b*_4_), i.e. T and G are swapped; otherwise *x*_3_ = *b*_4_. Observe that the mapping is not necessarily unique; however, to the best of our knowledge, the present one shows that it is possible to pass from the tessera code to the extant code by describing all the known degeneracy characteristics of the latter.

If, originally, protein coding involved codons longer than three bases, then the translation machinery should carry some memory of this. Indeed, the small subunit of extant ribosomes presents a structural freedom that could allow the inclusion of an additional nucleotide in the decoding centre so that the decoding of four-base codons is feasible. Note that the possibility of ancestral coding with quadruplets had been mentioned in [16]. Indeed, quadruplet decoding was discovered in 1973 [45] as a mechanism related to frameshift suppression and, nowadays, it is widely used in biotechnology applications in order to incorporate non-canonical amino acids into proteins [46–48]. Moreover, the biological feasibility of length-four codons and of an orthogonal ribosome that decodes them has been demonstrated in the laboratory [46]. Also, there is evidence that points to the existence of overlapping genes coded by tetracodons [49]; moreover, it has been shown that tetracodons play an important role in phylogenetic analysis, (e.g. [50]) and this can be an indication of a genetic memory.

An impressive number of properties of the tessera code is preserved in present codes. The early code and all its descendants inherit from the tessera code the number of codons (64 tesserae generate 64 codons) and the maximum number of amino acids (23). The tessera code allows to code for 24 elements/amino acids. Since at least one of these must represent a stop signal, the maximum theoretical number of representable amino acids is 23. Remarkably, no extant code exceeds this limit and the maximum number of amino acids that are coded directly by some genomes is exactly 23: the 20 standard amino acids plus 2 non-standard ones (selenocysteine and pyrrolysine) and the alternative initiation amino acid *N*-formylmethionine sum up to 23. Moreover, the number of adaptors used in the vertebrate mitochondrial genetic code is 22: eight tRNAs that recognize four codons each, 14 tRNAs that recognize two codons each, and two pairs of codons not associated with amino acids [34,51]. Remarkably, 22 is the absolute minimum observed among all known versions of the genetic code. Also, this is exactly the structure implied by the tessera model: eight primeval adaptors of degeneracy 4, plus 16 adaptors of degeneracy 2 form a set of 24 adaptors; if we discard two adaptors of degeneracy 2 assigned to stop codons we obtain exactly 22.

The vertebrate mitochondrial genetic code and our tessera-based model of the early code also share a number of features related to symmetry (e.g. table 5). First and foremost, the KM transformation, also known as Rumer’s transformation, applied to the first doublet of a codon changes the degeneracy of the corresponding amino acid. This universal property is observed in most known versions of the genetic code (both nuclear and mitochondrial). The tessera code also possesses this property. For example, the tessera AUUA corresponds to an amino acid of degeneracy 2, and if we apply the KM transformation to the first two nucleotides we obtain the tessera CGUA which corresponds to an amino acid of degeneracy 4. Note that this property also holds if we apply Rumer’s transformation to the *t*_12_ *t*_23_ of the mapping that connects tesserae and codons described above. For further insights, see [53].

**Table 5.** Comparative table between the vertebrate mitochondrial genetic code and the tessera code.

	vertebrate mitochondrial genetic code		tessera code	
	deg.	no. codons	deg.	no. tesserae
degeneracy	2	16	2	16
	4	8	4	8
number of codons	codons 64		tesserae 64	
number of adaptors	adaptors 22		adaptors 22	
number of amino acids	a.a. 20		a.a. 20	
symmetries				
Rumer	KM transform on the first two bases changes the degeneracy of the a.a.		KM transform on the first two bases changes the degeneracy of the a.a.	
Klein V group	the 16 codons sharing the transformation between the first and the second letter have the same degeneracy distribution		the 16 tesserae sharing the transformation between the first and the second letter have the same degeneracy distribution	

Another fundamental aspect of the tessera code is that the coding of a protein can be made robust to +1 frame shifts. The frame maintenance robustness can be also related to circular codes which have been hypothesized to play a role in the processes of frame synchronization [54–58]. The existence of a universal circular code property has been related to the origin of the genetic code as pairs of complementary codons coding for either the same or a similar amino acid [59]. The same conjecture is supported in other contexts [24]. This property arises naturally in the tessera code where a tessera and its reverse complement always code for the same amino acid.

## 4. Conclusion

The origin of degeneracy in protein coding has been described with minimal assumptions, indeed, only those regarding symmetry properties of coding and decoding ancient molecules and their possible stereo-chemical interactions. The theory is consistent with many attempts to describe the origin and evolution of degeneracy, for example, those regarding stereo-chemical recognition of long oligonucleotide sequences by ribozymes in a RNA world (see [36,60] and references therein), those regarding different symmetry approaches [1–3,8–10,12–14], and in particular, those claiming reverse recognition of codons [6]. In our approach, the description of the degeneracy of the pre-early genetic code is exact and does not arise as the result of ad hoc parameter tuning. Indeed, there are no free parameters in the model! Only symmetry properties matter: the fundamental status of symmetry principles in the physical sciences is brought to the same level of significance within molecular biology and evolution. The theory has many interesting implications, for example, the surprising result that the primitive and early versions of the genetic code are connected by an intermediate code (the pre-early code) with codons of length four and with a degeneracy distribution coincident with that of the present vertebrate mitochondrial genetic code. The evolutionary transitions between such ancestral codes are plausibly explained by evolutionary pressure related to decoding accuracy. Moreover, this scenario implies that some ancestral properties might have been preserved through evolutionary time scales. This is indeed the case, as we have shown, and their preservation poses a compelling challenge concerning their biological meaning. For example, one important question is how error correcting properties, that in the tessera code are explicitly identifiable, have been ‘translated’ in extant codes. Error control is an unavoidable requirement of any protein synthesis system. In extant genetic codes, the immunity to point mutations is no longer apparent. The matter of how extant decoding systems retain error correcting capabilities, in spite of the loss of redundancy due to codon length reduction, represents an interesting open question. In particular, the comparison between the tessera model and the mathematical model of the genetic code developed in [32,33,35,42,61,62] suggests that the role played by chemical transformations in the tessera model is mirrored by the role played by dichotomic classes in extant genetic codes and we will address the matter in future investigations.

The theory presented in this work is part of a unified mathematical framework that describes degeneracy with integer number representation systems (see box 1 and [33,61–63]). The framework is a new paradigm for interpreting genetic information and leads to the definition of mathematical objects that have a meaningful biochemical interpretation. For instance, dichotomic classes are binary variables derived from the model that are linked to the chemical properties of the nucleotides of a codon. The analysis of coding sequences of DNA by means of dichotomic classes highlighted the presence of universal strong short-range correlations that can be related to error detection [31,32]. Moreover, the theory of circular codes [52,54,56,58], a class of error detecting codes that have been proposed as strategies for frame detection and maintenance, can also be related to this mathematical framework [64].

Our work points to an origin of the degeneracy based on elementary properties of chemistry and physics, mainly the symmetries of primeval molecules. Moreover, our results suggest that both the symmetries and the degeneracy distribution of the mitochondrial code have been preserved through evolutionary times and prompt further fundamental questions.

## Supplementary Material

Supplementary Material

## Endnotes

1 For the English translation of the original articles of Rumer, see [30].

2 If we have two of these symmetries, the third one is automatically satisfied because it is the composition of the other two.

3 The second Chargaff parity rule postulates that, in each of the two DNA strands, there is a balance between the quantities of A and T and of G and C.

4 A point mutation of a tessera leads inevitably to a non-tessera.

5 Here, N_35_ denotes a generic nucleotide N in the 35th position of the tRNA. For instance, U_33_ denotes a uracil nucleotide in the 33rd position of the tRNA and corresponds to the nucleotide that precedes those in the anticodon positions 34, 35 and 36.

## Data accessibility

This article has no additional data.
