# Supplementary material for: On the origin of degeneracy in the genetic code
Source: Interface Focus. 2019 Oct 18;9(6):20190038. doi: 10.1098/rsfs.2019.0038 (PMC6802134; doi:10.1098/rsfs.2019.0038)
Supplement: Supplementary Material [file rsfs20190038supp1.pdf]

Supplementary Material for: On the origin of degeneracy in the genetic code

Diego Luis Gonzalez Simone Giannerini Rodolfo Rosa

September 4, 2019

A Chemical nucleotide transformations and symmetric groups

The genetic alphabet

$$\mathcal{B} := \{U(T), C, A, G\}$$

is composed of four letter or nucleotides: Uracil (Thymine), Cytosine, Adenine, and Guanine. The set of all possible bijective transformations over $\mathcal{B}$ has 24 elements and is defined as

$$S_{\mathcal{B}} = \{\pi : \mathcal{B} \to \mathcal{B} \mid \ \pi \textit{ is bijective}\}.$$

$S_{\mathcal{B}}$, together with the usual group operation given by composition as functions (denoted by $\circ$), forms a symmetric group on $\mathcal{B}$. The group $S_{\mathcal{B}}$ is isomorphic to the symmetric group S_4 on four elements (see **?** for more details). Hence, we write $\pi : (A, T, C, G) \to (G, A, T, C)$ if π satisfies $\pi(A) = G, \pi(T) = A, \pi(C) = T$, and $\pi(G) = C$. Bijective mappings $\pi : \mathcal{B} \to \mathcal{B}$ can be applied componentwise to $x \in \mathcal{B}^3$, the set of codons, and thus induce a bijective map $\mathcal{B}^3 \to \mathcal{B}^3$ which we denote also by π.

As shown in **?**, there are 4 bijective transformations that are invariant with respect to the chemical characters of the nucleotides. These are: Identity:

$$\mathrm{I} : (A, T, C, G) \to (A, T, C, G);$$

Strong/Weak (SW) or complementary transformation:

$$\mathrm{SW} : (A, T, C, G) \to (T, A, G, C);$$

Pyrimidine/Purine (YR) or parity transformation:

$$\mathrm{YR} : (A, T, C, G) \to (G, C, T, A);$$

and Keto/Amino (KM) or Rumer's transformation:

$$\mathrm{KM} : (A, T, C, G) \to (C, G, A, T).$$

In turn, these 4 transformations, together with the operation $\circ$ defined above, form a group on $\mathcal{B}^3$, which is isomorphic to the Klein V group and whose composition table is the following:

$\circ$	I	SW	KM	YR
I	I	SW	KM	YR
SW	SW	I	YR	KM
KM	KM	YR	I	SW
YR	YR	KM	SW	I

For instance, given the codon ACT, we can apply the two transformations SW and KM: SW(ACT) = TGA, KM(TGA) = GTC. Hence, (SW $\circ$ KM)(ACT) = GTC = YR(ACT).

B Jukes' code in a random world of tetracodons.

Jukes' code has 16 amino acids and 1 stop signal. Hence, we assume we have 17 objects/amino acids to be assigned randomly to $4^4 = 256$ tetracodons partitioned into 32 symmetric codons (with degeneracy 2) and 224 asymmetric codons (with degeneracy 4). Under this hypothesis, we compute the probability of observing a given degeneracy distribution. This corresponds to the hypergeometric probability of extracting without replacement k symmetric codons out of 17 objects, where the probability of extracting a symmetric codon is $p = 32/256 = 1/8$. We obtain:

$$P(X = k) = \frac{\binom{32}{k}\binom{224}{17-k}}{\binom{256}{17}}. \tag{1}$$

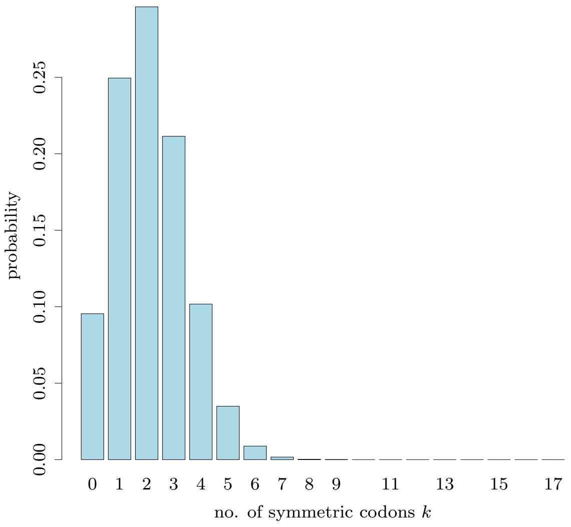

Figure 1: Hypergeometric probability function associated to choosing at random k symmetric elements/codons from 17. The mode of the distribution $k = 2$ corresponds to the degeneracy of Jukes' code and indicates that such code has maximal probability of being chosen if we assume a random assignation of tetracodons to amino acids.

The hypergeometric probability function above is presented in Figure 1. Clearly, the mode $k = 2$ corresponds to the degeneracy distribution of Jukes' code, that is, 2 elements of degeneracy 2 and 15 elements with degeneracy 4.
